# A Comparison of Online versus On-site Training in Health Research Methodology: A Randomized Study

**DOI:** 10.1186/1472-6920-11-37

**Published:** 2011-06-17

**Authors:** Rakesh Aggarwal, Nikhil Gupte, Nancy Kass, Holly Taylor, Joseph Ali, Anant Bhan, Amita Aggarwal, Stephen D Sisson, Sukon Kanchanaraksa, Jane McKenzie-White, John McGready, Paolo Miotti, Robert C Bollinger

**Affiliations:** 1 Sanjay Gandhi Postgraduate Institute of Medical Sciences, Lucknow 226014, India; 2 BJ Medical College Clinical Trials Unit, Pune, India; 3 Johns Hopkins University, Baltimore, Maryland, USA; 4 Researcher, Bioethics and Global Health, Pune, India; 5 Office of AIDS Research, NIH, Bethesda, Maryland, USA

## Abstract

**Background:**

Distance learning may be useful for building health research capacity. However, evidence that it can improve knowledge and skills in health research, particularly in resource-poor settings, is limited. We compared the impact and acceptability of teaching two distinct content areas, Biostatistics and Research Ethics, through either on-line distance learning format or traditional on-site training, in a randomized study in India. Our objective was to determine whether on-line courses in Biostatistics and Research Ethics could achieve similar improvements in knowledge, as traditional on-site, classroom-based courses.

**Methods:**

*Subjects: *Volunteer Indian scientists were randomly assigned to one of two arms.

*Intervention: *Students in Arm 1 attended a 3.5-day on-site course in Biostatistics and completed a 3.5-week on-line course in Research Ethics. Students in Arm 2 attended a 3.5-week on-line course in Biostatistics and 3.5-day on-site course in Research Ethics. For the two course formats, learning objectives, course contents and knowledge tests were identical.

*Main Outcome Measures: *Improvement in knowledge immediately and 3-months after course completion, compared to baseline.

**Results:**

Baseline characteristics were similar in both arms (n = 29 each). Median knowledge score for Biostatistics increased from a baseline of 49% to 64% (p < 0.001) 3 months after the on-site course, and from 48% to 63% (p = 0.009) after the on-line course. For the on-site Research Ethics course, median score increased from 69% to 83% (p = 0.005), and for the on-line Research Ethics course from 62% to 80% (p < 0.001). Three months after the course, median gains in knowledge scores remained similar for the on-site and on-line platforms for both Biostatistics (16% vs. 12%; p = 0.59) and Research Ethics (17% vs. 13%; p = 0.14).

**Conclusion:**

On-line and on-site training formats led to marked and similar improvements of knowledge in Biostatistics and Research Ethics. This, combined with logistical and cost advantages of on-line training, may make on-line courses particularly useful for expanding health research capacity in resource-limited settings.

## Background

There is great need and demand for building health research capacity globally, to ensure that opportunities and competence to undertake scientifically and ethically sound research exist in all regions [1,2]. Current strategies to train investigators in clinical research skills rely on traditional class-room teaching that is expensive to provide and difficult to scale up in resource-limited settings.

Distance learning, including on-line teaching, may provide an attractive, cost-effective, scalable and efficient alternative to on-site classroom training. On-line education formats use diverse media such as text, images, audio, video and interactive formats [3]. On-line learning has been widely utilized for education in the US and other developed country settings [4]. In these settings, much research has been undertaken to assess the effectiveness of online training in diverse fields. These studies have shown that well-designed online medical education courses result in knowledge gains similar to, and at times superior to, traditional classroom teaching [5,6]. In addition, several courses have also shown evidence of significant self-reported practice change [7]. Further, more recently, various online training formats have been compared to identify those that offer the best opportunities for imparting e-learning in terms of participant satisfaction and gains in knowledge [8]. In addition, several guidelines and reviews on the subject of e-learning and design of content for such training have been published [9-11].

Distance education has also supported training of students in resource-limited settings. India has the largest distance learning university in the world, offering nearly 350 courses with approximately 2.8 million students currently enrolled [12,13]. However, evaluations of the impact and acceptance of on-line learning platforms in such settings are limited. In addition, there is limited information on the ability of distance learning to improve knowledge and skills relevant for health research.

To better understand the potential value of on-line learning platforms to expand health research capacity, we undertook a randomized study comparing on-line with on-site (i.e. face-to-face) delivery of information in two distinct domains relevant for international health research: Biostatistics and Research Ethics. Further, to assess the feasibility and potential of utilizing on-line platforms for expanding health research capacity in resource-limited settings, we conducted this study in India. Our hypothesis was that both on-site and on-line course formats would lead to similar gains in knowledge for students, for both content domains. We further hypothesized that trainees would report a similar level of satisfaction for on-line and on-site course platforms.

## Methods

### Participants

Volunteers for the study were recruited through an announcement in Indian biomedical journals and via email invitations to individuals engaged in biomedical research, and leaders of medical schools and major institutions conducting health research in India. The eligibility criteria for inclusion were: (i) a degree in medicine or a masters' degree in science, (ii) receipt of a graduate or postgraduate degree within the last ten years, (iii) at least one-year of experience in human health-related clinical or social science research, (iv) basic computer skills and availability of broadband internet access, (v) willingness to be randomized and to participate in the study, and (vi) willingness to undertake pre- and post-course evaluations. Interested persons were invited to register at a website, and respond to questions relevant to eligibility. Four investigators (two in India [RA, AA] and two in the US [PM, RB]) reviewed the applications.

### Randomization and Study Procedures

**Figure 1:**
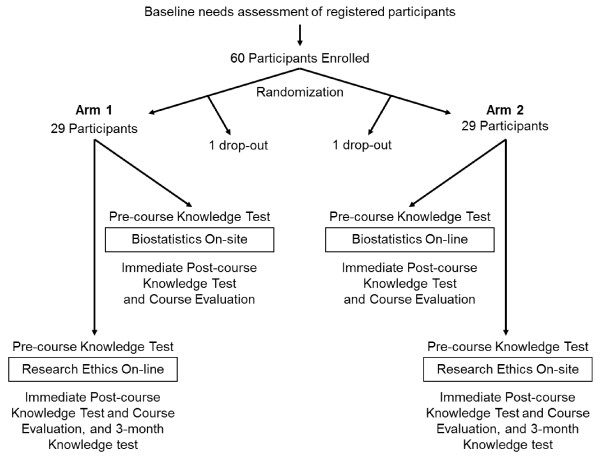
**A summary of procedures used in the study**.

The study used a randomized design. Following informed consent, each study participant was allocated to one of two study arms, using a computer-based randomization procedure. Participants in Arm 1 traveled to Lucknow, India for a 3.5-day classroom training in Biostatistics, and a week later participated from their own home or office settings in a 3.5-week on-line training course in Research Ethics (Figure 1). Those in Arm 2 received a 3.5-week on-line training in Biostatistics and then traveled to Lucknow for a 3.5-day on-site training in Research Ethics. Courses were provided at no cost to participating students, and all travel, accommodation, and meal costs for participation in the on-site courses were provided. Participants were not otherwise compensated financially or in kind for loss of time. Before attending the Biostatistics and Research Ethics courses, each participant completed a short course about on-line learning methods; this also helped confirm that they had adequate computer hardware and internet bandwidth required for effective participation in on-line courses.

### Course Descriptions

Content for all courses was developed using established principles of curriculum development [14]. Learning objectives, course materials and course lecture slides were identical for the on-line and on-site formats of both the Biostatistics and Research Ethics courses. For the on-line courses, students viewed slide presentations while listening to lectures pre-recorded by faculty experts. Adobe Presenter software program was used to produce the synchronized lecture and slide presentation. Students were provided with readings to accompany or complement each lecture, as well as copies of all lecture slides (PDF files for on-line course; printouts for on-site course). Both the Biostatistics and Research Ethics on-line and on-site courses included structured live group activities and case discussions, in addition to the formal lectures. On-line students also had the option of posting questions through a web portal, to facilitate discussion with fellow students and course faculty. On-site courses included time for questions and discussion during and after lectures.

The Biostatistics course was developed by JM, based on an introductory level course he developed for the Department of Biostatistics at Johns Hopkins Bloomberg School of Public Health, with input on learning objectives, course content and knowledge assessments contributed by faculty experts from India (RA, NG and AA). The Biostatistics course included 17 lectures over 15.5 hours, that covered statistical analysis and study design, including types of data, descriptive statistics, normal distribution, sampling distribution, central limit theorem, confidence intervals, hypothesis testing for continuous and categorical data, simple linear regression, measures of association, survival analysis, and design of observational and interventional studies. Students also participated in 8 interactive group exercise sessions of 45-60 minutes each designed to apply knowledge gained during the lectures. On-line students were provided the same exercises to discuss and solve on-line with other students and the course faculty, during 8 interactive sessions. All lectures for the on-line format were pre-recorded by JM. He was unable to travel to India for the on-site course, and the three Indian faculty experts in biostatistics (RA, NG and AA) delivered lectures, using the same slides as those used in the on-line course. They interacted via email with JM and repeatedly listened to his on-line lectures, in order to deliver the on-site live lectures consistent with JM's on-line lectures. The 8 interactive sessions for both the on-line and on-site formats were conducted by two Indian faculty experts (RA, NG).

The Research Ethics course was developed by faculty experts at the Johns Hopkins Berman Institute of Bioethics (NK and HT), with input on learning objectives, course content and knowledge assessments contributed by a faculty expert from India (AB). The three Research Ethics faculty delivered 15 lectures with 8.75 hours of instruction, covering ethical principles, a framework for ethical analysis, informed consent, the relationship of study design to ethics, risk/benefit assessment, the role of ethics committees, privacy/confidentiality, and honesty in science. In addition, students participated in 5 one-hour interactive case discussions, requiring application of ethical analysis skills. Students also viewed and discussed one 20-minute video on ethical challenges in community-based research. Course participants completed two homework assignments. The same faculty experts from the US and India provided the lectures and cases discussions for both the on-line and on-site formats of the Research Ethics courses.

The interactive sessions for online courses were conducted at pre-specified times and lasted about 60 minutes each. Each session was moderated by one or more course faculty. The course participants were encouraged to log in into these sessions over the Internet, though attendance was not compulsory. During these online 'classrooms', faculty could use audio to address student questions asked via a typed message that appeared on the faculty members computer screen as well as the screen of all other students. The faculty could also share his/her computer screen, on which s/he could write and draw. Further, the moderator could give 'audio' rights to any student, allowing him/her to speak to the entire class.

For the statistics online course, eight online sessions were so arranged that each topic was covered in two consecutive sessions to allow a participant who could not join a particular session to 'attend' the other; each participant was therefore expected to attend four sessions. The participants were encouraged to send to moderators any questions that they wanted discussed. The faculty member reviewed the question and answers for these with the students.

The research ethics faculty held five case-based discussions with the students in the on-line course. Three sessions were held for each case-based discussion, each moderated by a member of the faculty. Students were given the case and a set of questions to answer in advance of the session. The faculty member would review the question and answers with the students and then moderate a discussion on the case.

### Outcome Measures

For each training course (on-site or on-line, Biostatistics or Research Ethics), all participants were administered knowledge tests before the course, as well as immediately and three months after course completion. These tests focused on assessment of participants' knowledge related to the specific learning objectives and application of this knowledge to problems raised in case histories. Responses were scored against pre-determined answers, and an overall knowledge score (percent correct responses) was computed for each test. Gains in knowledge scores in each domain (Biostatistics or Research Ethics) were compared between the participants receiving on-site and on-line training. The tests of knowledge were the same for on-line and the on-site platforms in each content domain. The biostatistics knowledge tests consisted of 20 questions in objective format (single response, true/false, or providing answers based on simple calculations). The research ethics knowledge assessments given just before and after the course were unique sets of 41 multiple choice, true/false and short answer questions. The three month follow-up knowledge assessment consisted of the 41 best performing questions from the pre and immediate post-course assessments.

The gain in knowledge at 3 months after completion of each course was used as the primary outcome measure. In addition, at the completion of each course, each study participant was administered a course evaluation questionnaire to assess student acceptability and satisfaction with the course. This assessment utilized a 5-point Likert scale for most items; for a few questions, 4-point and 3-point Likert scales were used.

### Ethical Review

The study was approved by ethics committees at both the participating institutions (Institutional Ethics Committee of the Sanjay Gandhi Postgraduate Institute of Medical Sciences, Lucknow, India and Johns Hopkins Medical Institutions Institutional Review Board, Baltimore, Maryland, USA). Each study participant signed a written informed consent prior to randomization, and submitted it by fax, as scanned computer file by email or through postal mail.

### Statistical Analysis

Wilcoxon's rank sum test and Wilcoxon's signed rank test were used for inter-group and paired comparisons, respectively. Multivariate quartile regression analyses were used to determine independent predictors of gain in knowledge scores at 3 months following course completion, in each domain. Covariates, including age, number of years since last degree, and mode of training (on-site versus online), were tested in univariate analyses as predictors of gain in knowledge scores in biostatistics and in research ethics separately. Those found significant at p < 0.05 level were included in a multivariable analysis. Data on acceptability of and satisfaction with each course were compared using a trend test for ordinal data. In addition, the effect of demographic factors on the relationship between gain in knowledge at 3 months from baseline and the training platform (on-line or on-site) was examined using a quartile regression analysis; factors found significant on univariate analysis were entered into a multivariate analysis. Data on acceptability of and satisfaction with each course and mode of instruction were compared using a chi-squared test for trend for ordinal data.

## Results

### Baseline Characteristics of Students

A total of 250 persons registered on-line for participation in the study, and 75 volunteers were invited to submit a completed and signed consent form. Of these, 60 invited volunteers agreed to participate, submitted signed consent forms and were randomized to either Arm 1 or Arm 2. Two volunteers, one randomized to each arm, subsequently elected to drop out of the study prior to initiation, due to inability to travel on the dates of the on-site course or to attend the on-line course, respectively. Thus, a total of 58 volunteers participated. Their median age was 34 years [Range 25-48 years] and 45 (78%) were male. Participants in each arm were similar in age, gender distribution, number of years since obtaining last degree, and pre-training baseline knowledge scores in both Biostatistics and Research Ethics domains (Table 1). One volunteer in Arm 1 did not complete any of the post-course knowledge assessments in Research Ethics, and 3-month post-course assessment in Biostatistics, and one in Arm 2 did not complete the 3-month post-course knowledge assessment in Biostatistics.

**Table 1:** Baseline Student Characteristics

Characteristic	Arm 1 On-site Biostatistics, On-line Research Ethics (n = 29)	Arm 2 On-line Biostatistics, On-site Research Ethics (n = 29)
Age, years (range)	36 (28-39)	34 (30-38)

Male gender, n (%)	23 (79)	22 (76)

Current position is institutional faculty, n (%)	15 (52%)	15 (52%)

Baseline knowledge score, % correct (range)		
Biostatistics	49 (16-79)	48 (14-79)
Ethics	62 (40-89)	69 (36-89)

All data are shown as median (range) or actual numbers, as appropriate.

Comparisons were done using Wilcoxon's rank sum test or chi-squared test.

There was no significant difference between participants randomized to Arm 1 and Arm 2 for any of the variables.

### Knowledge Gains

Biostatistics knowledge scores, assessed immediately after the course, showed a significant increase from baseline, for both the on-site (p < 0.001) and on-line course (p = 0.009) formats (Table 2). When assessed again 3-months after completion of the course, the median Biostatistics knowledge scores were significantly higher than baseline for both on-line and on-site formats. For students randomized to the on-site Biostatistics course format (Arm 1), the median knowledge score increased from 49% at baseline to 64% at 3-months post-course (p = 0.005). For students randomized to the on-line Biostatistics course (Arm 2), the median knowledge score increased from 48% to 63% (p = 0.005).

**Table 2:** Knowledge at Baseline, Immediately and 3 Months after Completion of Biostatistics and Ethics Courses.

Knowledge Domain	Time point	On-site Format Median (Range)	On-line Format Median (Range)	p value
**Biostatistics**	Pre-course	49% (16-79)	48% (14-79)	0.95
	
	Immediately after Course Completion	74% (17-100)*	58% (38-80)**	0.004
	
	Three months after Course Completion	64% (20-85)***	63% (28-85)***	0.78
	
	Median Knowledge Gain 3 Month Post Course vs. Baseline Assessment	16% (-17% to 49%)	12% (-10% to 65%)	0.59

**Human Research Ethics**	Pre-course	69% (36-89)	62% (40-89)	0.07
	
	Immediately after Course Completion	82% (44-93)*	77% (43-95)***	0.02
	
	Three months after Course Completion	83% (54-98)*	80% (32-100)*	0.69
	
	Median Knowledge Gain 3 Month Post Course vs. Baseline Assessment	17% (0% to 41%)	13% (-15% to 34%)	0.14

All data are shown as median (range).

All statistical comparisons were done using Wilcoxon's rank sum test.

*p < 0.001, **p = 0.009, ***0.005 compared to pre-course scores for the same domain and format

Research Ethics knowledge scores also increased significantly from baseline, for both the on-site and on-line platforms (Table 2; p < 0.001 and p = 0.005, respectively). At 3-months after completion of the courses, the median Research Ethics knowledge scores were significantly higher than baseline for both on-line and on-site formats. For students randomized to the on-line Research Ethics course (Arm 1), the median knowledge score increased from 62% at baseline to 80% at 3-month post-course evaluation (p < 0.001). For students randomized to the on-site Research Ethics course (Arm 2), the median knowledge score increased from 69% at baseline to 83% at 3-month post-course (p < 0.001).

### Comparison of On-line Training and On-site Training

When assessed immediately following course completion, the median gain in Biostatistics knowledge compared to baseline was higher among students participating in the on-site course format (28% median knowledge gain), than those in the on-line course format (11% median knowledge gain; p = 0.02) (Table 2). However, when knowledge of Biostatistics was assessed again 3 months after course completion, the increase in knowledge compared to baseline was similar among students in the on-site and on-line formats (16% gain and 12% gain, respectively; p = 0.59). When assessed immediately following course completion, the median gain in Research Ethics knowledge compared to baseline was similar among students participating in the on-site course format (15% median knowledge gain), compared to students participating in the on-line course format (10% median knowledge gain; p = 0.19). The increase in Research Ethics knowledge compared to baseline, was sustained when assessed 3 months after course completion and similar for both on-site and on-line formats (17% and 13% median knowledge gain, respectively; p = 0.14).

### Effect of Demographic Characteristics on Knowledge Gain

For the Biostatistics Course, neither the type of instructional platform nor any covariate (i.e. baseline characteristics including age, gender, faculty status or baseline knowledge score) was significantly associated with knowledge gain on univariate analysis (data not shown). However, knowledge gain in Research Ethics was significantly associated with younger age and lower baseline knowledge scores on univariate analysis. On multivariate analysis, only lower pre-course knowledge score was independently associated with a greater gain in Research Ethics knowledge (0.25, p-value = 0.002). For both univariate and multivariate analyses, instructional platform (on-line vs on-site) was not associated with significant differences in knowledge gain for Research Ethics (data not shown).

### Student Satisfaction

Overall, a very high level of student satisfaction ("Agree" or "Strongly Agree") was demonstrated for both on-site and on-line formats, and for both Biostatistics (Tables 3, 4 and 5) and Research Ethics courses (Tables 6, 7 and 8). For Biostatistics, the students ranked the interactive sessions and some course instructor performance characteristics during the on-site course higher than those for the on-line course; this difference was observed for 5 of the 14 characteristics included in the questionnaire (Tables 3, 4). For Research Ethics courses, on-site course scored significantly better than on-line course for 2 of the 18 characteristics studied; in particular, the students ranked the case study discussions during the on-site course higher than those during the on-line course (Tables 6, 7). In addition, on-line participants were more likely than on-site participants to report that the pace of the Research Ethics course was "too fast" (p < 0.001) (Table 8).

**Table 3:** Student Satisfaction with the On-site and On-line Biostatistics Courses (Section 1)

Variable	Arm 1 On-site Biostatistics					Arm 2 On-line Biostatistics					p value*
		
	1	2	3	4	5	1	2	3	4	5	
The course content matched the defined course objectives	0	0	1	14	15	0	0	2	11	15	0.909

The course materials improved/enhanced learning of the subject	0	0	4	11	15	0	0	1	13	14	0.722

**The live talks enhanced learning in courses**.	**0**	**0**	**3**	**8**	**19**	**0**	**1**	**14**	**8**	**5**	**0.001**

The course was organized in an appropriate way to learn the concepts outlined in the course objectives	0	0	1	15	14	0	0	4	18	6	0.025

**Constructive feedback was given during live talks**	**0**	**0**	**3**	**15**	**12**	**0**	**2**	**11**	**11**	**4**	**0.001**

Course topics were related to the main ideas in the course	0	0	1	15	14	0	0	0	18	10	0.505

Class materials related to the broader context of biomedical research	0	1	0	13	16	0	0	2	18	8	0.059

The course will be valuable to my work in biomedical research	0	0	0	9	21	0	0	1	10	17	0.413

The course instructors explained the material clearly.	0	0	1	9	20	0	0	1	16	11	0.048

The course instructors held my interest during the course	0	0	0	8	22	0	0	4	12	12	0.010

**The course instructors held my interest during the course**	**0**	**0**	**3**	**8**	**19**	**0**	**2**	**10**	**11**	**5**	**< 0.001**

**The course instructors were accessible/approachable**	**0**	**0**	**1**	**2**	**27**	**0**	**0**	**5**	**12**	**11**	**< 0.001**

1 = strongly disagree; 2 = disagree; 3 = somewhat agree; 4 = agree; 5 = strongly agree

*p values are based on comparisons using chi-squared test for trend. Data with p values below 0.05 are shown in bold.

**Table 4:** Student Satisfaction with the On-site and On-line Biostatistics Courses (Section 2)

Variable	Arm 1 On-site Biostatistics				Arm 2 On-line Biostatistics				p value*
		
	1	2	3	4	1	2	3	4	
**Overall, the course instructors were..**.	**0**	**0**	**5**	**25**	**0**	**0**	**15**	**13**	**0.003**

Ability of faculty to resolve and respond to course related concerns or problems was...	1	0	10	18	0	1	16	9	0.056

1 = below average; 2 = average; 3 = good; 4 = excellent

*p values are based on comparisons using chi-squared test for trend. Data with p values below 0.05 are shown in bold.

**Table 5:** Student Satisfaction with the On-site and On-line Biostatistics Courses (Section 3)

Variable	Arm 1 On-site Biostatistics			Arm 2 On-line Biostatistics			p value*
		
	Too slow	Just right	Too fast	Too slow	Just right	Too fast	
The speed at which the material was covered in the course was...	0	23	7	0	17	11	0.193

The time allocated for reviewing the formal lectures in this course was...	5	24	1	9	19	0	0.128

The time allocated to the live talk sessions of the course topics was...	4	26	0	3	19	6	0.057

*p values are based on comparisons using chi-squared test for trend. Data with p values below 0.05 are shown in bold.

**Table 6:** Student Satisfaction with the On-site and On-line Research Ethics Courses (Section 1)

Variable	Arm 1 On-line Research Ethics					Arm 2 On-site Research Ethics					P value*
		
	1	2	3	4	5	1	2	3	4	5	
The course content matched the defined course objectives	0	0	1	14	15	0	0	0	7	21	0.046

Materials for the course improved/enhanced learning of the subject.	0	0	1	16	13	0	0	1	8	19	0.077

The case studies were helpful in understanding and applying the ethical principles.	0	0	2	8	20	0	0	0	8	20	0.589

The movie was a helpful tool for understanding and applying principles and concepts of informed consent.	0	1	10	10	9	0	1	3	9	15	0.038

The framework was a helpful tool for understanding and applying ethical principles.	0	0	1	11	18	0	0	1	6	21	0.250

**Group Discussion was a helpful tool for understanding and analyzing the issues that arose in the case studies**.	**0**	**0**	**4**	**11**	**15**	**0**	**0**	**0**	**4**	**24**	**0.003**

The course was organized in an appropriate way to learn the concepts outlined in the course objectives.	0	2	3	9	16	0	0	0	12	16	0.419

Timely feedback was given on assignments.	0	1	3	14	12	0	0	1	12	15	0.197

Constructive feedback was given on assignments.	0	1	4	12	13	0	0	1	13	14	0.344

Course topics were related to the main ideas in the course.	0	0	3	12	15	0	0	0	11	17	0.275

This class was relevant to the broader context of biomedical research.	0	0	1	12	17	0	0	1	6	21	0.165

**This course will be valuable to my work in biomedical research**.	**0**	**0**	**2**	**11**	**17**	**0**	**0**	**0**	**3**	**25**	**0.005**

The course instructors explained the material clearly.	0	0	2	12	16	0	0	0	12	16	0.618

The course instructors held my interest during the course.	0	1	2	14	13	0	0	2	12	14	0.568

The course instructors challenged and motivated me to learn in this course.	0	2	5	11	12	0	0	1	12	15	0.108

The course instructors were accessible/approachable.	0	0	2	15	13	0	0	1	9	18	0.114

1 = strongly disagree; 2 = disagree; 3 = somewhat agree; 4 = agree; 5 = strongly agree

*p values are based on comparisons using chi-squared test for trend. Data with p values below 0.05 are shown in bold.

**Table 7:** Student Satisfaction with the On-site and On-line Research Ethics Courses (Section 2)

Variable	Arm 1 On-line Research Ethics				Arm 2 On-site Research Ethics				P value*
		
	1	2	3	4	1	2	3	4	
Overall, the course instructors were...	0	1	10	19	0	0	8	20	0.469

Ability of faculty to resolve and respond to course related concerns or problems was	0	3	11	16	0	0	9	18	0.211

1 = below average; 2 = average; 3 = good; 4 = excellent

*p values are based on comparisons using chi-squared test for trend. Data with p values below 0.05 are shown in bold.

**Table 8:** Student Satisfaction with the On-site and On-line Research Ethics Courses (Section 3)

Variable	Arm 1 On-line Research Ethics			Arm 2 On-site Research Ethics			P value*
		
	Too slow	Just right	Too fast	Too slow	Just right	Too fast	
**The speed at which material was covered in the course was..**..	**0**	**20**	**10**	**5**	**23**	**0**	**< 0.001**

The time allocated to each Group Discussion was.....	7	23	0	2	26	0	0.092

*P values are based on comparisons using chi-squared test for trend. Data with p values below 0.05 are shown in bold.

## Discussion

Our study comparing on-line to on-site teaching formats in a resource-limited setting demonstrated that both formats significantly increased knowledge from baseline and this increase in knowledge was observed for both content areas (Biostatistics and Research Ethics). In addition, the increases in knowledge were sustained for 3 months after completion of the courses. There was a high level of student satisfaction for all courses, although the on-site format was associated with a somewhat higher level of satisfaction related to instructor accessibility and quality of faculty feedback.

A recent meta-analysis of 126 on-line learning interventions demonstrated that all but two of these were associated with a gain in knowledge [15]. Most of these 126 studies were from developed countries and three evaluated the impact of courses related to building health research capacity (including one each on research methodology, statistics and institutional review board policies). Our study from India demonstrated significant improvements in knowledge after on-line courses in a resource-poor setting for two quite disparate research training domains, a finding consistent with those in resource-rich setting.

In another meta-analysis covering the impact of on-line training in diverse fields from school level to professional level [4], on-line training was associated with a higher gain in knowledge. However, the difference was very limited when analysis was restricted to studies that compared face-to-face and pure on-line training formats. In comparison, a much larger gain in knowledge was seen when blended (a mixture of on-line and face-to-face) instruction programs and face-to face programs were compared [4], though even these effects were considered to be related to differences in time spent in learning, curriculums and pedagogy than to differences in training formats. Thus, our findings at 3-months after on-site training and on-line training that included interactive sessions in a developing country setting mirror the findings from resource-rich settings. In this context, it is important to note we used scheduled group e-learning sessions, in which the participants could log-in and discuss the course material with other participants and facilitators; this format has previously been shown to perform better than an on-demand format where such discussion sessions were not scheduled [8].

Although results were similar when assessed 3 months after the courses, for both knowledge domains, we found higher knowledge scores, particularly for Biostatistics, immediately after the on-site courses, than after the on-line course. Several factors may account for this observation. First, our study participants may not have had internet access of reasonable quality. Although we verified each participant's access to sufficient broadband internet connectivity at the beginning of the study, intermittent outages may have occurred. Second, participants in a resource-limited setting may not be as familiar with on-line courses as in US universities where 20-25% of all students take at least one on-line course [3]. Another explanation may be related to the work pattern of physicians in developing countries. Since such physicians, who constituted a majority of our study subjects, typically spend a larger share of their time on clinical duties than those in industrialized countries, they may find it difficult to spend time reading and reviewing on-line course material during a routine day. In contrast, during an on-site course, being away from their institutions and clinical work may have permitted the participants to focus better on the training activity. In the previously cited meta-analysis [4], the better outcome of on-line training was considered to have resulted from the on-line trainees spending more time on training activities than that spent during face-to-face courses, rather than to on-line delivery format *per se*. Alternatively, the difference may be related to better learning during on-site training, possibly due to the influence of factors such as face-to-face rather than screen-to-screen interaction with the instructor.

The difference in immediate knowledge gain between the on-line and on-site formats was more marked for Biostatistics, than for Research Ethics. A possible explanation of this difference is that the quantitative skills required for Biostatistics may be poor in medical professionals. Greater faculty-trainee interaction during on-site training may thus be helpful when training medical professionals in this domain. The results of the participant satisfaction survey, in which the on-site course scored higher in questions related to faculty-trainee interaction, would support this explanation. The greater satisfaction with on-site courses may also mean that further efforts are needed to enhance faculty-trainee interaction during on-line training.

Our study has several potential limitations. First, participants in our study may not be adequately representative of the population of prospective biomedical researchers that require training in research methodology. Our study participants may have been highly motivated, and therefore likely to have greater gains in knowledge scores with any type of training. However, due to the randomized design, this effect would have been similar for on-line and on-site courses, and would not affect our conclusions of comparative performance of the two training formats. Another potential limitation is that our study participants were from India and our results may not be generalizable to other populations with less access to and familiarity with the internet. Finally, although both the on-line and on-site Research Ethics courses used the same faculty, this was not possible for the two Biostatistics courses. However, in a previous meta-analysis, it was shown that a change in faculty member did not influence effectiveness of an on-line course, provided the course content and method of delivery were unchanged [4].

Another limitation of our study, designed as a comparative efficacy trial of two formats of training, was the inability to accurately assess the costs per person for each training format. Attendance at a course entails several different types of costs, including course fees, costs of travel and accommodation, and costs due to lost wages or work, etc; further, the course fees include costs of faculty time, course material, and facilities used. Ours being a research study, travel and hotel accommodation for all participants were arranged on a uniform scale, and these had no relationship with the costs that the participants would have incurred if they had arranged and paid for these. Because we used some of the pre-existing course materials and online facilities, costs of development of new courses for either on-site or online training courses could not be assessed accurately. Also, for the on-site ethics course, faculty members travelled from USA to India; this expenditure is unlikely to occur in real-life. Further, the 'cross-over' study design limited the number of participants in the online courses, precluding true assessment of per capita costs. Thus, in view of the unusual settings, we could not compare comparison of costs incurred per capita for the two training formats.

A training program can be evaluated at various levels. A popular approach to evaluation of training, the Kirkpatrick evaluation model, delineates four levels of learning outcomes [16,17]. These include: (i) Reaction (assessment of participants' reaction to and satisfaction with the training program); (ii) Learning (degree of increase in intended knowledge, skills, attitudes and confidence); (iii) Behavior (application of learnt knowledge and skills once trainees are back on the job); and, (iv) Results (degree of targeted outcomes in terms of effect on business, efficiency, monetary terms, etc). Our study assessed only the first two domains. The remaining higher-level domains, though more important, cannot be easily assessed in an efficacy study of the kind we undertook. Assessment of those domains is possible only with long-term training programs, and further studies on the role of online training in attaining improvement in these domains are warranted.

On-line teaching provides several advantages over more traditional on-site course formats, particularly for building research capacity in resource-limited settings. On-line courses can be more flexible, convenient and accessible [9,18], particularly for busy clinicians in communities where there is a shortage of health care providers and allow interactivity and adaptability to individual learner styles [5,9,11]. Also, such courses can be accessed by a much larger number of persons, from diverse geographic locations. This scalability is likely to offset the time and cost investment required for the on-line course development and for the interactive components. Although preparation of on-line training material may require a high initial financial investment, the recurring costs of such training are generally lower, because faculty and student travel costs are eliminated, and faculty time required for delivering didactic course material, though not for interactive sessions, is reduced in the long run.

## Conclusion

In our randomized controlled study, On-line and on-site training formats led to marked and similar improvements of knowledge in Biostatistics and Research Ethics. Since on-line training has several logistical and cost advantages over on-site training, distance learning using on-line tools may be a particularly useful, efficient, cost-effective and scalable strategy for expanding health research capacity in resource-limited settings.

## List of abbreviations used

US: United States; USA: United States of America;

## Competing interests

The authors declare that they have no competing interests.

## Authors' contributions

RA, NG, NK, HT, AB, AA, SDS, JM, PM and RCB were involved in conception and design of the study. RA, NG, HT, JA, AB, AA, SDS, SK, JM, JMW, RCB played a role in acquisition of data. RA, NG, HT, JA, AB, AA, RCB were involved in analysis and interpretation of data. The initial manuscript draft was prepared by RA, AA, JMW and RCB. All the authors participated in critical revision of manuscript for intellectual content. NG did the statistical analysis. RA and RCB played a role in obtaining funding for the study. RA, NG, JA, AB, AA, SK, JMW and RCB provided administrative, technical or material support. RA, NG, AA, SK, PM and RCB supervised the study. All the authors have read and approved the final manuscript. RA and RCB had full access to all of the data in the study and take responsibility for the integrity of the data and the accuracy of the data analysis. The sponsor played no role in data analysis or in the decision to publish this paper.

## Pre-publication history

The pre-publication history for this paper can be accessed here:

http://www.biomedcentral.com/1472-6920/11/37/prepub
